# Dynamics of Vacancy Formation and Distribution in Semiconductor Heterostructures: Effect of Thermally Generated Intrinsic Electrons

**DOI:** 10.3390/nano13020308

**Published:** 2023-01-11

**Authors:** Timur S. Shamirzaev, Victor V. Atuchin, Vladimir E. Zhilitskiy, Alexander Yu. Gornov

**Affiliations:** 1 Laboratory of Physics and Technology of Heterostructures, Institute of Semiconductor Physics, SB RAS, Novosibirsk 630090, Russia; 2 Department of Applied Physics, Novosibirsk State University, Novosibirsk 630090, Russia; 3 Laboratory of Optical Materials and Structures, Institute of Semiconductor Physics, SB RAS, Novosibirsk 630090, Russia; 4 Research and Development Department, Kemerovo State University, Kemerovo 650000, Russia; 5 Department of Industrial Machinery Design, Novosibirsk State Technical University, Novosibirsk 630073, Russia; 6 R&D Center “Advanced Electronic Technologies”, Tomsk State University, Tomsk 634034, Russia; 7 Optimal Control Laboratory, Institute for System Dynamics and Control Theory, SB RAS, Irkutsk 664033, Russia

**Keywords:** vacancy generation, recombination, semiconductor heterostructure, diffusion

## Abstract

The effect of thermally generated equilibrium carrier distribution on the vacancy generation, recombination, and mobility in a semiconductor heterostructure with an undoped quantum well is studied. A different rate of thermally generated equilibrium carriers in layers with different band gaps at annealing temperatures forms a charge-carrier density gradient along a heterostructure. The nonuniform spatial distribution of charged vacancy concentration that appears as a result of strong dependence in the vacancy formation rate on the local charge-carrier density is revealed. A model of vacancy-mediated diffusion at high temperatures typical for post-growth annealing that takes into account this effect and dynamics of nonequilibrium vacancy concentration is developed. The change of atomic diffusivity rate in time that follows on the of spatial vacancy distribution dynamics in a model heterostructure with quantum wells during a high-temperature annealing at fixed temperatures is demonstrated by computational modeling.

## 1. Introduction

The high-temperature post-growth annealing of heterostructures is useful for the engineering of semiconductor devices because it allows band-gap structure modification [1,2,3,4,5,6,7,8,9] and an oscillator strength [10,11], controls hyperfine interaction [12], adjusts the dephasing time of excitons [13], and reduces strain gradients [14,15,16,17,18,19]. For III-V semiconductor heterostructures, the changes occurring at the annealing are based on the intermixing that proceeds via the vacancy-mediated mechanism in which the atom jumps into a vacancy induced by high-temperature heating at a neighboring lattice site [2,3,20,21]. Thus, the vacancy generation probability and diffusivity factor (*D_V_*) play key roles in post-growth annealing processes. It is well known that vacancies in semiconductors can be electrically charged [7,8,9,10,11,12,13,14,15,20,21,22,23,24,25,26,27,28,29]. As a result, a strong dependence of the vacancy formation energy on the charge-carrier concentration occurs [4,23,24,28,29,30,31]. The effect of thermally generated equilibrium carriers on vacancy-related phenomena was well studied in bulk crystals or thick semiconductor layers with a uniform carrier distribution [25,28,29,32].

In semiconductor heterostructures that contain layers with different band gaps, in contrast to the bulk materials, there is a dispersion in the rate of the thermally generated equilibrium carriers for different layers. The redistribution of these carriers between the layers with temperature and annealing time can modify the spatial distribution of vacancy generation and diffusion rates during the annealing time. However, the effect of thermally generated carrier distribution in a semiconductor heterostructure on the vacancy generation and diffusion during the high-temperature annealing has been scarcely studied so far.

In this paper, the effect of thermally generated carrier distribution on the vacancy generation and recombination probabilities in an undoped heterostructure with quantum wells (QWs) is studied theoretically and by computational modeling. We demonstrate that a change in the vacancy generation and recombination rates in the QW region can occur during high-temperature annealing as a result of an increase or decrease in the concentration of thermally generated electrons during the annealing at a fixed temperature. The dynamics of nonequilibrium vacancy concentration was also taken into account. Thus, nonlinearity in the vacancy-mediated atomic diffusion in time occurs near the heterojunction region at high-temperature annealing, even when neglecting the vacancy-native defect complex formation at a high vacancy concentration [32,33]. The effects discussed here are particularly important for semiconductors heterostructures with vacancy-mediated atomic diffusion such as Ge, Si, III-V, and II-VI compounds. Although the used one-dimensional model has obvious limitations (calculations were made for QWs), our main goal is to demonstrate the effect of thermally generated equilibrium carrier distribution on vacancy generation and diffusion, which is relevant for a full dimensional model. We also note that the phenomena revealed in our study are very general and take place at high temperatures for the formation, recombination, and migration of any charged defects. The obtained results can be applied to any low-dimensional semiconductor heterostructure.

In order to simplify the further analysis, we will use the following approximations:

(1) In the general case, vacancies are formed in different ways. The main paths are generation at the surface (Schottky defect formation) and generation in the volume (Frenkel pair formation). In the first case, to consider the vacancy formation, it is necessary to take into account the state of the surface (e.g., the coating type and structure [34,35,36,37,38]) and the free surface exchange by atoms and molecules with the environment [39,40,41,42]. For most semiconductor materials, spontaneous Frenkel pair formation is considered an unlikely source of vacancies thanks to the high energy of forming two defects as a tight pair. However, here we focus on the processes occurring deep in the material volume only. Therefore, we neglect the defect formation and recombination at the surface (Schottky defects) and the defect diffusion from and to the surface. Thus, in our case, the vacancy concentration is determined by the temperature-activated formation of Frenkel pairs (vacancy + interstitial atom).
(2) The recombination of Frenkel pairs occurs only when the interstitial atom is located in the nearest internode to the vacancy (short-range interaction limit).
(3) Vacancies can be neutral or have different charge states (negative for certainty), while the interstitial atom is neutral. The general case, where both the vacancy and interstitial atom have charge states, is discussed in Appendix A.
(4) We neglect the vacancy participation in the complex defect formation, such as bi-vacancies and other complexes of point defects.
(5) We also neglect a change of the vacancy formation and migration energies with a change in the composition of semiconductor alloys that takes place in the QW vicinity during diffusion [43]. Thus, we have a similar probability for the jump of vacancy to the host and the impurity atom states.

## 2. Vacancy in Uniform Crystals

Let us first briefly consider the well-known case of uniform bulk crystal. In this case, the vacancy *N_V_* distribution can be described by the following kinetic equation:

(1) $$\frac{\partial N_{V}(t)}{\partial t}=G(t)-R(t),$$

where *G* is a vacancy generation rate and *R* is a vacancy recombination rate. Note that both *G* and *R* can be changed with time. The vacancy generation rate is the Arrhenius function $A\exp\left(-H_{A}/kT\right)$, where *A* is a pre-exponential factor, *H_A_* is an activation enthalpy, and *k* is the Boltzmann constant. The pre-exponential factor can be written as follows:

(2) $$A=\gamma_{c}N_{AP}N_{IP}\nu \exp\left[\frac{S_{f}+S_{m}}{k}\right],$$

where *N_AP_* is the number of atoms that can go to an interstitial place with a vacancy formation; *N_IP_* is the number of interstitial places near *N_AP_* atoms; ν is the Debye frequency; *S_f_* and *S_m_* are the formation entropy and the migration entropy, respectively; and *γ_c_* is a coefficient that depends on the interaction mechanism between a vacancy and an interstitial atom. In the general case, it needs to take into account that vacancies can be in differently charged states [23,25,28,29,32,44], and both *H*_A_ and *A* values depend on the charged vacancy state.

### 2.1. Neutral Vacancy Formation

Neutral vacancy formation produces a change only in the atomic subsystem of a crystal, because any atom in the crystal lattice can go to the interstice with the formation of a vacancy *N_AP_* = *N_IP_* = *N*, where *N* is the atoms density in an appropriate sublattice and the pre-exponential factor is governed by the equation $A^{0}=\gamma_{c}N^{2}\nu \exp\left[(S_{f}+S_{m})/k\right]$. The activation enthalpy of the neutral vacancy formation is $H_{A}^{0}=H_{f}^{0}+H_{m}^{0}$, where $H_{f}^{0}$ is a formation enthalpy that reflects the energy costs of the atomic system for the vacancy and interstitial atom formation and $H_{m}^{0}$ is a migration enthalpy that shows the energy costs for vacancy or interstitial atom migration that results in the spatial separation of Frenkel defects [32,44]. Note that, typically, interstitial migration has lower migration barriers compared to vacancies [45]. Therefore, we relate $H_{m}^{0}$ to the interstitial atom migration. Thus, the neutral vacancy formation rate can be written as follows:

(3) $$G_{}^{0}=\gamma_{c}N^{2}\nu \exp\left[\frac{S_{f}+S_{m}}{k}\right]\exp\left[-\frac{H_{f}^{0}+H_{m}^{0}}{kT}\right].$$

The activation energy for Frenkel pair recombination contains, in a short-range interaction limit, an energy barrier of $H_{m}^{0}$. Therefore, the vacancies recombination rate can be written as follows:

(4) $$R_{}^{0}=R_{0}\exp\left[-\frac{H_{m}^{0}}{kT}\right],$$

where $R_{0}=\gamma_{r}N_{V}^{0}N_{I}^{}\nu \exp\left[\frac{S_{m}}{k}\right]$, $N_{I}$ is the interstitial atom concentration, and $N_{V}^{0}$ is vacancy concentration. In the case of bulk crystal with the uniform defect distribution, $N_{V}^{0}=N_{I}$ occurs. Factor γ*_r_* can be written as γ*_r_* = σ*r*, where σ is the annihilation cross section of an interstitial atom with a vacancy and *r* is the vacancy and interstitial atom interaction radius [29]. In the short-range interaction limit for a Frenkel pair, we can estimate *r* = *a* and σ = *a*^2^, where *a* is a lattice constant.

In the thermodynamic equilibrium, the neutral vacancy concentration is described by a well-known expression:

(5) $$N_{V}^{0}=N\exp\left[\frac{S_{f}^{}}{2k}\right]\exp\left[-\frac{H_{f}^{0}}{2kT}\right].$$

Because this equation stems from the solution of Equations (1), (3), and (4), we can find in this approximation that γ*_c_*= *a*^3^.

Capturing a charge carrier can charge a neutral vacancy. However, this trivial case, when a vacancy manifests itself like a regular donor or acceptor, does not affect the probability of Frenkel pair formation and is not interesting for our study. In the next subsection, we consider the direct charged vacancy formation.

### 2.2. Charged Vacancy Formation

**Figure 1:**
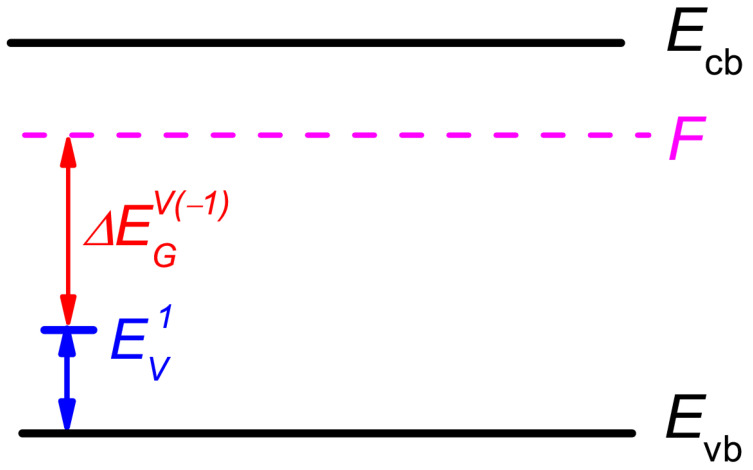
Schematic energy diagram of a single-charged vacancy. Vertical arrows: blue—charged vacancy ionization energy; red—energy gap between vacancy ionization energy and Fermi level (dashed magenta).

The formation of a charged vacancy modifies both the atomic and electronic subsystems of the crystal [23,24,25]. To be certain, we will consider negatively charged vacancies that capture electrons at the formation stage. In this case, the pre-exponential factor is corrected to a probability of an electron to be at the spatial point of the vacancy formation:

(6) $$A^{\left(-j\right)}=a^{3}N_{AP(-j)}N_{IP(-j)}\nu \exp\left[\frac{S_{f}+S_{m}}{k}\right],$$

where *N_AP(−j)_* is the number of atoms that can go to an interstitial place and have, in the immediate vicinity, an electron that can be captured by a vacancy during the formation. *N_IP(−j)_* is the number of interstitial places near *N_AP(−j)_* atoms. In a bulk crystal, the following equality occurs: *N_AP(−j)_* = *N_IP(−j)_* = *N_−j_*. Factor *j* reflects the vacancy charge state. It can be equaled, for example, to 0, 1, 2, and 3 for a vacancy charged with zero, one, two, and three electrons, respectively. We skip the charge state sign + or − when this coefficient is a degree. The activation enthalpy changes in the following manner: $H_{A}^{j}=H_{A}^{0}+\Delta E_{G}^{V(-j)}$. This additional energy is $\Delta E_{G}^{V(-j)}=\sum_{i=1}^{j}\left(E_{V}^{i}-F\right)$, where *F* is the Fermi-level position and $E_{V}^{i}$ is the ionization energy of vacancy with charge −*i* [23,25,28], as it is schematically shown in Figure 1 for the single-charged vacancy. Here and in what follows, all energies are measured with respect to the valence band top (*E*_vb_). Therefore, we can write the vacancies generation rate in *j* charged state as follows:

(7) $$G_{}^{-j}=a^{3}N_{-j}^{2}\nu \exp\left[\frac{S_{f}+S_{m}}{k}\right]\exp\left[-\frac{H_{A}^{0}+\sum_{i=0}^{j}E_{V}^{i}-jF}{kT}\right]$$

In this equation and in what follows, we denote $E_{V}^{0}\equiv 0$.

To be at the spatial point of the vacancy formation, an electron probability must equal the ratio of the free electron concentration (*n*) to the conduction band density of states (*N_ce_*) [40]. Thus, the equality *N_AP(−j)_* = *N_IP(−j)_* = *N_−j_* = *N*(*n*/*N_ce_*) occurs. In the case of undoped material, the equilibrium electron density can be written as [46] follows: $n=N_{ce}\exp[(F-E_{cb}^{})/kT]$, where *E_cb_* is the conduction band edge energy. Therefore, we can write the following for undoped crystals: $N_{-j}^{2}=N^{2}{\left(n/N_{ce}\right)}^{2}=N^{2}\exp[2(F-E_{cb})/kT]$. Note that in this case, we have the following relation for the pre-exponential factor: $A^{\left(-j\right)}=A^{0}{\left(n/N_{ce}\right)}^{2j}$. Finally, the general equation for the −*j* charged vacancy formation rate in undoped materials has the following form:

(8) $$G_{}^{-j}=a^{3}N_{}^{2}\nu \exp\left[\frac{S_{f}+S_{m}}{k}\right]\exp\left[-\frac{H_{A}^{0}+\sum_{i=0}^{j}E_{V}^{i}+j(2E_{cb}-3F)}{kT}\right].$$

At first sight, a term that contains 3*F* in the exponent of this equation looks like we have taken into account three electrons in the vacancy formation process. However, it just reflects the fact that both the activation energy and the amount of crystal cells and interstitials, in which a Frenkel pair containing a charged vacancy can be formed, are a function of electron concentration. One value of Fermi level, *F*, contributed in this factor comes from the activation energy, and the 2*F* contribution comes from probability to find the crystal cell containing an electron and the neighboring interstitial (that has the same physical meaning as *N*^2^ in Equation (2)).

According to Equation (8), the change in the formation probability of a vacancy with charge −*j*, with respect to the neutral one, is determined by a factor $\Delta_{j}=j(2E_{cb}-3F)+\sum_{i=1}^{j}E_{V}^{i}$, which is a function of the Fermi-level position that depends on the electron concentration. This probability is increased for a negative Δ*_j_* and decreased for a positive Δ*_j_*. In other words, under the Δ*_j_* > 0 condition, the neutral vacancy formation probability will exceed that for negatively charged ones, and vice versa for Δ*_j_* < 0.

**Figure 2:**
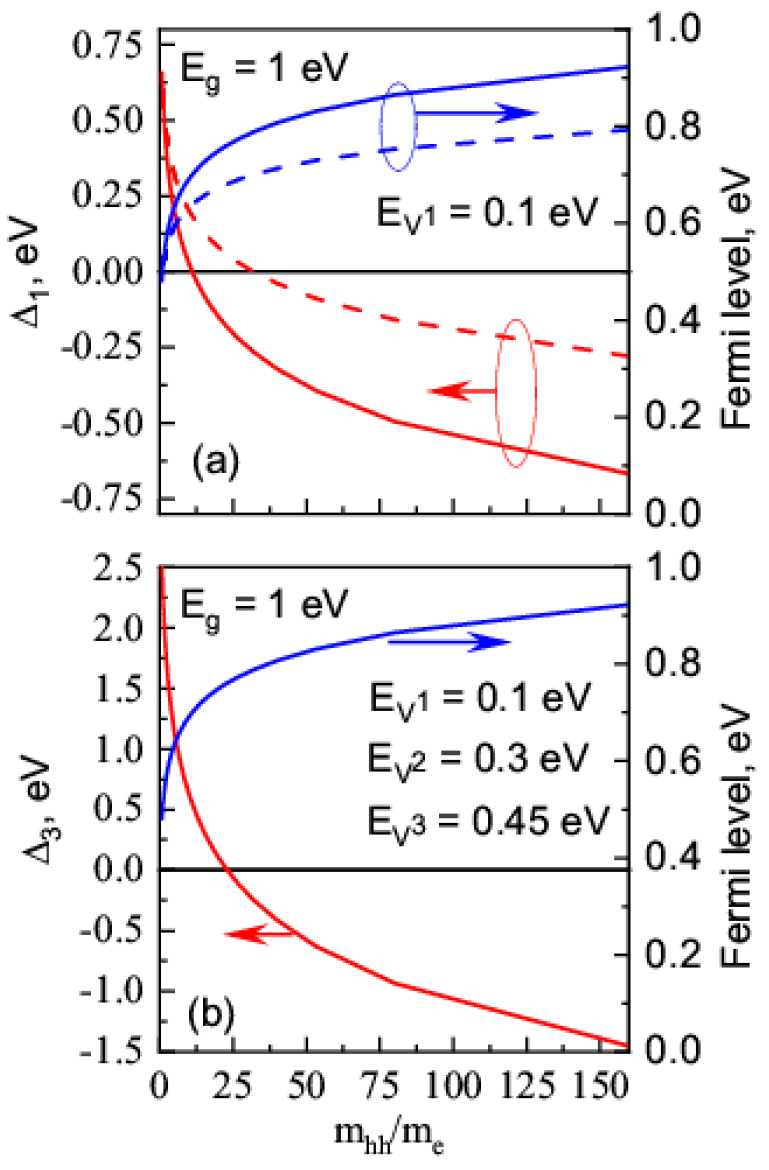
Factor Δ*_j_* (red) and the Fermi-level position (blue) calculated as functions of heavy hole to effective electron mass ratio, for the material with *E_g_* = 1 eV. (**a**) Single-charged vacancy with electron ionization energy $E_{V}^{-1}$ = 0.1 eV for *T* = 1300 K (solid lines) and for 900 K (dashed lines); (**b**) triple-charged vacancy with the ionization energy for the first, second, and third electron, $E_{V}^{-1}$ = 0.1 eV, $E_{V}^{-2}$ = 0.3 eV, and $E_{V}^{-3}$ = 0.45 eV, respectively, for *T* = 1300 K.

Because $F=E_{cb}/2+(3/4)kT\ln(m_{hh}^{}/m_{e})$, where *m_hh_* and *m_e_* are the effective mass of a heave hole and electron, respectively [46], we can easily estimate the condition when Δ*_j_* < 0 is realized. Factor Δ*_j_* and the Fermi-level position calculated as functions of heavy hole to electron effective masses ratio for single- and triple-charged vacancies are shown in Figure 2. One can see that the Fermi-level position corresponding to Δ*_j_* < 0 should be shifted into the conduction band direction that can be realized in materials with the constraint $m_{e}<<m_{hh}$.

For the vacancy recombination rate, we can put it down as previously:

(9) $$R_{}^{j}=R_{j}\exp\left[-\frac{H_{m}^{0}}{kT}\right],$$

where $R_{j}=a^{3}N_{V}^{-j}N_{I}^{}\nu \exp\left[\frac{S_{m}}{k}\right]$. Thus, the concentration equilibrium in an isolated system of -*j* charged vacancies is as follows:

(10) $$N_{V}^{-j}=N\exp\left[\frac{S_{f}^{}}{2k}\right]\exp\left[-\frac{H_{A}^{0}+\sum_{i=0}^{j}E_{V}^{i}+j(2E_{cb}-3F)}{2kT}\right].$$

### 2.3. Vacancy Recharging

Vacancies in a crystal are formed in various charged states. After their formation, the vacancies can be recharged. The following system of kinetic equations describe the dynamics of neutral and single-charged vacancies:

(11) $$\begin{array}{l} \begin{array}{l} \frac{\partial N_{V}^{0}(t)}{\partial t}=a^{3}N^{2}\nu \exp\left[\frac{S_{f}+S_{m}}{kT}\right]\exp\left[-\frac{H_{f}^{0}+H_{m}^{0}}{kT}\right]-a^{3}N_{V}^{0}(t)N_{I}^{}(t)\nu \exp\left[\frac{S_{m}}{k}\right]\exp\left[-\frac{H_{m}^{0}}{kT}\right]-\gamma N_{V}^{0}(t)+\\ \gamma N_{V}^{-1}(t)\exp\left[\frac{F(t)-E_{V}^{-1}}{kT}\right]; \end{array}\\ \begin{array}{l} \frac{\partial N_{V}^{-1}(t)}{\partial t}=a^{3}N^{2}\nu \exp\left[\frac{S_{f}+S_{m}}{kT}\right]\exp\left[-\frac{H_{f}^{0}+H_{m}^{0}}{kT}\right]\exp\left[-\frac{E_{V}^{-1}+2E_{cb}-3F(t)}{kT}\right]+\gamma N_{V}^{0}(t)-\\ \gamma N_{V}^{-1}(t)\exp\left[\frac{F(t)-E_{V}^{-1}}{kT}\right]-a^{3}N_{V}^{-1}(t)N_{I}^{}(t)\nu \exp\left[\frac{S_{m}}{k}\right]\exp\left[-\frac{H_{m}^{0}}{kT}\right]; \end{array}\\ N_{I}^{}(t)=N_{V}^{0}(t)+N_{V}^{-1}(t) \end{array}$$

where $\gamma =a^{3}N_{ce}/\tau$ is a normalized trapping coefficient of an electron for a neutral vacancy (τ is a trapping time), and *N_vh_* is a valence band density of states. The change in charged carrier concentration also occurs with the creation of charged vacancy. Therefore, one has to complete the system of Equation (11) via neutrality Equation (12), which takes into account the dynamics of electron and hole concentration in the crystal [29,40].

(12) $$N_{ec}\exp\left[\frac{F(t)-E_{cb}^{}}{kT}\right]+N_{vh}\exp\left[-\frac{F(t)}{kT}\right]+\frac{N_{V}^{-1}(t)}{1+\exp\left[-E_{V}^{-1}/kT\right]}=0.$$

In order to demonstrate the vacancy concentration dynamics, we took the vacancy parameters of a widely used semiconductor, GaAs. The enthalpy of formation and that of migration for Ga vacancies in GaAs are still the subject of a debate [22,23,26,27,47,48,49,50,51,52,53,54]. We collect some gallium vacancy parameters in GaAs (proposed in the literature values of entropies and enthalpies for the neutral vacancy formation and migration, electron ionization energy in different charged states) in Table 1. One can see that for the formation enthalpy, the values are from 2.8 up to 3.8 eV, and for the migration enthalpy, they are from 0.8 up to 3.3 eV. Other material parameters used in calculations were taken from [55]. It is shown that the Ga vacancy is found to exist in 0, −1, −2, and −3 charge states [26,51]. However, we will here use a simple model that contains neutral and single-charged vacancies only that is just enough to qualitatively demonstrate the effect of equilibrium electrons on the creation of a vacancy in a system with more or less realistic parameters.

**Figure 3:**
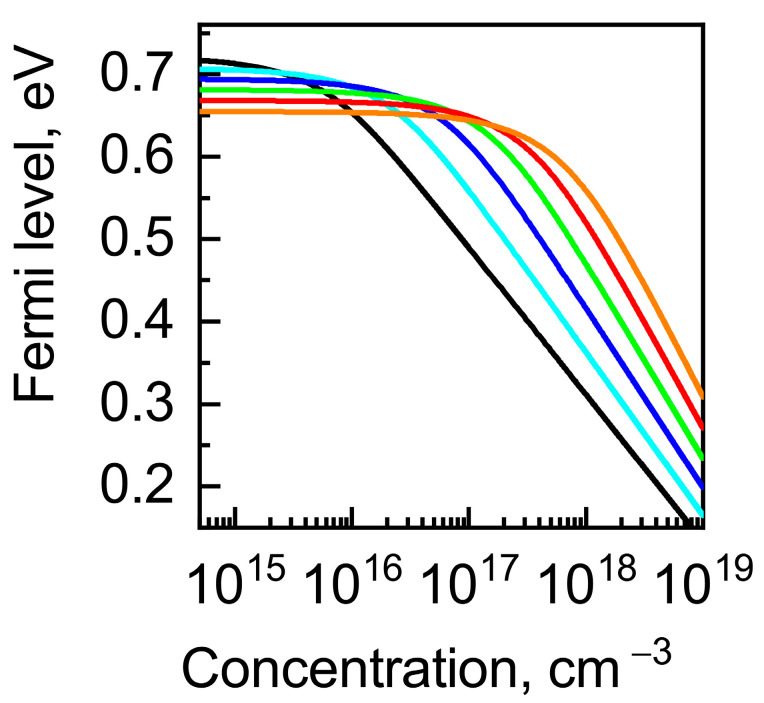
Fermi-level energy calculated as a function of charged vacancy concentration for different temperatures, K: 900 (black), 1000 (cyan), 1100 (blue), 1200 (green), 1300 (red), and 1400 (orange).

First of all, we calculated the charged vacancy formation effect on the Fermi-level position that reflects the change in the charge-carrier concentration in the crystal with the used neutrality Equation (12). The result of the calculations for different temperatures is shown in Figure 3. One can see that there are two charged vacancy concentration regions: (i) a region where the Fermi level does not depend on the vacancy concentration (i.e., the intrinsic concentration of electrons exceeds that of the charged vacancy) and (ii) a region where the Fermi level decreases with increasing vacancy concentration (i.e., the charged vacancy concentration exceeds that of the intrinsic electrons). The boundary between these regions depends on the temperature.

**Figure 4:**
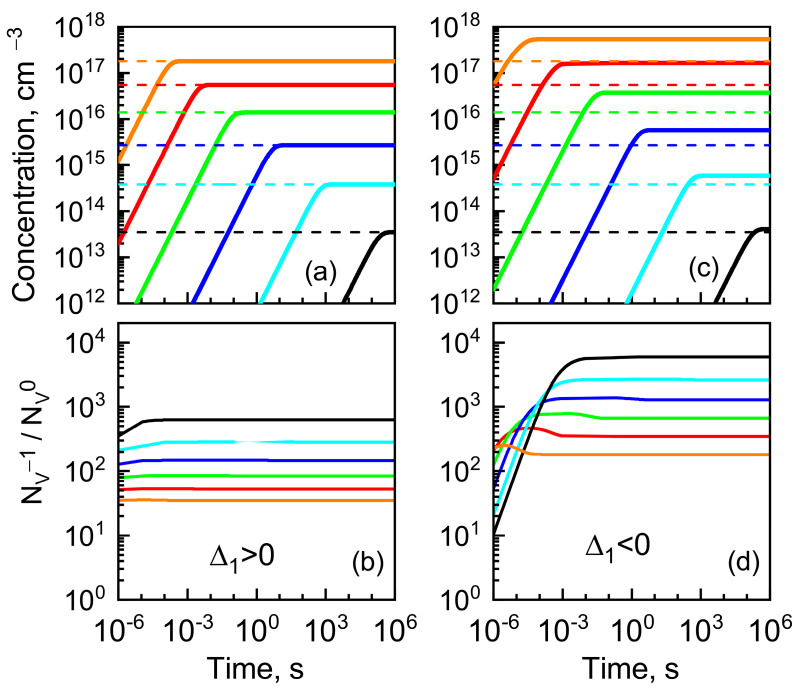
Dynamics of charged vacancy concentration (**a**,**c**) and ratio of charged to neutral vacancy concentrations (**b**,**d**) calculated for different temperatures: 900 K (black), 1000 K (cyan), 1100 K (blue), 1200 K (green), 1300 K (red), and 1400 K (orange), with $H_{f}^{0}$ = 3.7 eV and $H_{m}^{0}$ = 2.7 eV for cases (**a**,**b**) Δ_1_ > 0 (*m_hh_*/*m_e_* = 4) and (**c**,**d**) Δ_1_ < 0 (*m_hh_*/*m_e_* = 50). Colored dashed lines related to the corresponding temperature mark of the equilibrium concentration of neutral vacancy.

The dynamics of charged vacancy concentration and the ratio of charged to neutral vacancy concentrations energy were calculated for different temperatures, with $H_{f}^{0}$ = 3.7 eV and $H_{m}^{0}$ = 2.7 eV for the cases Δ_1_ > 0 and Δ_1_ < 0. The effect of charged vacancy concentration on the Fermi-level position (see Figure 3) was taken into account in the calculation. The calculation results are shown in Figure 4.

The vacancy concentration dynamics is a typical rising curve with saturation. One can see that in both cases, charged vacancies dominate over neutral ones. However, for the case of Δ_1_ > 0, the saturation level corresponds to the equilibrium concentration of the neutral vacancy. Therefore, the vacancies appear mainly as a result of the formation of a neutral Frenkel pair, and it captures an electron after the separation of the pair. At the same time, for the case of Δ_1_ < 0, the charged vacancy concentration exceeds the equilibrium concentration of neutral vacancies; i.e., the formation of charged vacancies (formation of charged Frenkel pair) is more efficient than that of neutral ones. This scenario will be in the focus of our subsequent analysis.

The calculation results allow one also to conclude that in bulk a material, (i) the equilibrium concentration of Frenkel vacancy is achieved only in special cases at high temperatures or in long annealing times, and (ii) the vacancy concentration is not at a thermal equilibrium value and depends on the preliminary temperature treatment of the sample. For example, after heating a heterostructure during the growth procedure, the vacancy concentration remains nonequilibrium after cooling. Thus, vacancies generated at the crystal growth do not have time to fully recombine during cooling. This conclusion is in the best agreement with numerous experimental data [21,56,57,58].

## 3. Spatial Distribution of Vacancies in a Heterostructure with QWs

**Figure 5:**
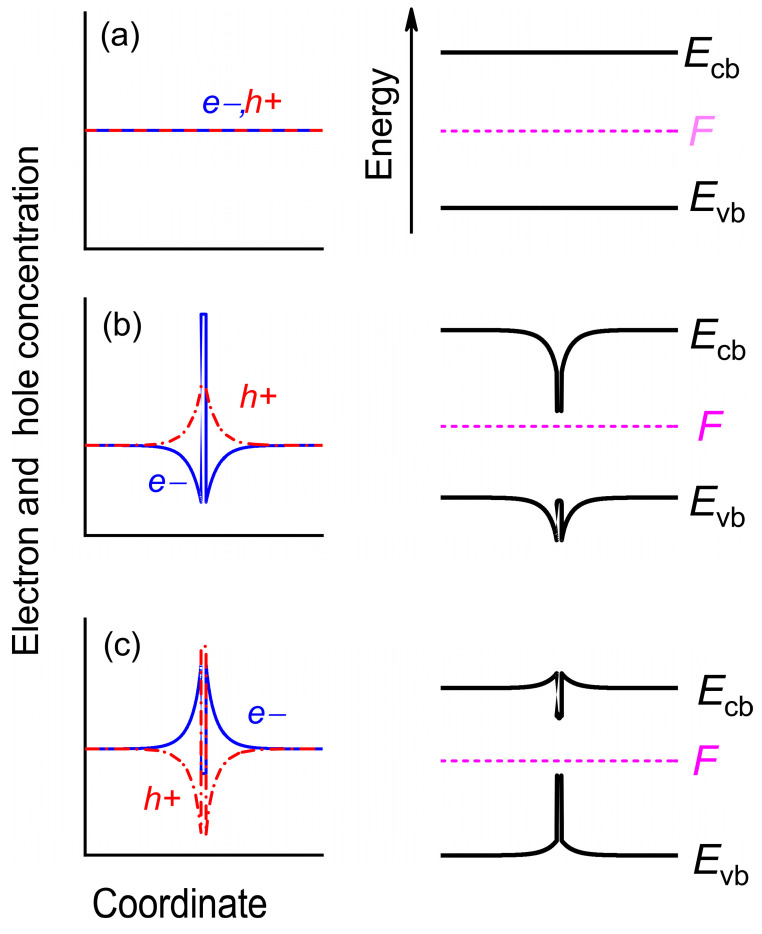
Schematic band diagrams and spatial electron e− and hole h+ distributions. (**a**) Bulk material, (**b**) QW with Fermi level shifted toward the conduction band, (**c**) QW with the Fermi level shifted toward the valence band.

A semiconductor heterostructure with QWs can be described as a thin layer of a low band-gap semiconductor material embedded between thick layers of another one with a higher band-gap (matrix). The principal difference between a bulk semiconductor and a heterostructure with QWs is spatial charge distribution, as it is shown in Figure 5. Far away from the surface in a bulk semiconductor, the equilibrium charge distribution is setting according to the Fermi-level position, and it is uniform (Figure 5a). The charge vacancy generation rate is proportional to the electron concentration that results in the uniform spatial distribution of the vacancy. The situation is drastically changed in a heterostructure with QWs. First of all, the concentration of equilibrium electrons and holes in a QW increase, with respect to the bulk matrix, thanks to the smaller band gap of the QW material. Additionally, away from the QW, the Fermi-level position is determined by the bulk semiconductor parameters. However, inside the QW, the Fermi-level position does not follow the parameters of QW material, but rather, it depends on the conduction and valence band offset. When a large band offset fraction occurs in the conduction (valence) band, the Fermi-level energy is shifted toward the QW conduction (valence) band, while the equilibrium electron and hole concentration follow the Fermi level $n=N_{ec}\exp[(F-E_{cb}^{})/kT]$, $p=N_{vh}\exp[(E_{vh}^{}-F)/kT]$ [46].

Notwithstanding system electroneutrality on the whole, a local redistribution of charge carriers occurs within the heterostructure, and the equilibrium concentration of electrons around the QW does not equal that of holes, which is schematically illustrated in Figure 5b,c. Note that this effect manifests itself at high temperatures when there is a noticeable charge-carrier generation.

One can see in these figures that the nonuniform carrier distribution leads to the band’s banding. The energy distances between Fermi level and the conduction band bottom, as well as the vacancy ionization energy that affects the activation energy for the creation of charged vacancy (see Equation (6)) begin to be a function of coordinates. Therefore, the spatial vacancy distribution becomes nonuniform. In this case, one has to take into account the vacancy diffusion along the heterostructure. The energy balance at the vacancy diffusion can be calculated as the energy change at a vacancy recombination and its creation in the neighboring state. For the diffusion without charge variation, the energy change just equals the vacancy migration enthalpy $H_{mv}^{0}$. The diffusivity factor can be written as $D_{V}=D_{V}^{0}\exp[-H_{mv}^{0}/kT]$, where $D_{V}^{0}=ga^{2}\nu \exp(S_{m}/k)$ with a factor *g* that is completely determined by the atomic jumps geometry (*g* = 1 for GaAs [47]).

In the case of diffusion with a charge variation (that can be present as the recombination of a vacancy with *m* electrons and its creation with the *l* one), there is an additional change in the electronic subsystem energy equal to $\Delta E_{ml}^{}=\sum_{i=1}^{l}\left(F-E_{V}^{i}\right)-\sum_{i=1}^{m}\left(F-E_{V}^{i}\right)$. Therefore, the diffusivity factor is rewritten as $D_{V}=D_{V}^{0}\exp[-(H_{mv}^{0}+\Delta E_{ml}^{})/kT]$. Noteworthy is that at a negative $\Delta E_{ml}^{}$ (increase in the amount of the electrons at the vacancy after a jump) vacancy completely charges in a few jumps and that further diffusion proceeds without charge variation. Positive $\Delta E_{ml}^{}$ (decrease in the amount of the electrons at vacancy after a jump) also hampers the diffusion with a charge variation.

Neglecting the vacancy charge variation during the diffusion, one can rewrite the system of Equations (11) and (12) as follows:

(13) $$\begin{array}{l} \begin{array}{l} \frac{\partial N_{V}^{0}(z,t)}{\partial t}=a^{3}N^{2}\nu \exp\left[\frac{S_{f}+S_{m}}{kT}\right]\exp\left[-\frac{H_{f}^{0}+H_{m}^{0}}{kT}\right]-a^{3}N_{V}^{0}(z,t)N_{I}^{}(z,t)\nu \exp\left[\frac{S_{m}}{k}\right]\exp\left[-\frac{H_{m}^{0}}{kT}\right]\\ -\gamma N_{V}^{0}(z,t)+\gamma N_{V}^{-1}(z,t)\exp\left[\frac{F(t)-E_{V}^{-1}(z,t)}{kT}\right]+D_{V}\left[\frac{\partial^{2}N_{V}^{0}(z,t)}{\partial z^{2}}\right]; \end{array}\\ \begin{array}{l} \frac{\partial N_{V}^{-1}(z,t)}{\partial t}=a^{3}N^{2}\nu \exp\left[\frac{S_{f}+S_{m}}{kT}\right]\exp\left[-\frac{H_{f}^{0}+H_{m}^{0}}{kT}\right]\exp\left[-\frac{E_{V}^{-1}(z,t)+2E_{cb}(z,t)-3F(t)}{kT}\right]+\gamma N_{V}^{0}(z,t)-\\ \gamma N_{V}^{-1}(z,t)\exp\left[\frac{F(t)-E_{V}^{-1}(Z,t)}{kT}\right]-a^{3}N_{V}^{-1}(z,t)N_{I}^{}(z,t)\nu \exp\left[\frac{S_{m}}{k}\right]\exp\left[-\frac{H_{m}^{0}}{kT}\right]+D_{V}\left[\frac{\partial^{2}N_{V}^{-1}(z,t)}{\partial z^{2}}\right]; \end{array}\\ N_{ec}\exp\left[\frac{F(t)-E_{cb}^{}(z,t)}{kT}\right]+N_{vh}\exp\left[-\frac{F(t)}{kT}\right]+\frac{N_{V}^{-1}(z,t)}{1+\exp\left[-E_{V}^{-1}(z,t)/kT\right]}=0. \end{array}$$

Here one has to take into account the following features of the system: (i) The Fermi-level position is the same along the heterostructure, and it is determined by the processes occurring in a bulk semiconductor far away from a QW. (ii) The local generation rate of charged vacancy depends on a coordinate owing to the band bending in the region around a QW. The band bending affects both the local electron concentration and local activation energy in that both $E_{cb}^{}$ and $E_{V}^{-1}$ shift with respect to the Fermi level. (iii) The energies of conduction band and valence band edge, as well as the electron concentration in the region around a QW, change with time. The first one is due to the material intermixing during the low band-gap material atom diffusion into the matrix layers; the second follows the band-gap change and, additionally, is due to the charged vacancy formation. Both of these processes result in the band-bending dynamics. Thus, in order to obtain a self-consistent system of equations describing the vacancy distribution dynamics, it is necessary to supplement Equation (13) in the following way: to take into account the diffusion of the low band-gap material atoms in the thick matrix layers (e.g., the dynamics of *C*(*z,t*) that is the spatially dependent function of material composition, the fraction of low band-gap material atoms in the matrix) and to add the equation $E_{cb}^{}(z,t)=f(C(z,t))$ that describes the effect of the energy spectrum modification around the QW in the diffusion process.

It is well known that the interdiffusion process in III-V heterostructures is governed by the vacancy diffusion on a given sublattice [2,20]. Atoms jump over to the place of vacancy in the neighboring lattice site. When the diffusion is dominated by the vacancy mechanism, the diffusivity factor is proportional to the density of vacancy *N_V_* [21]. In this case, one can take into account that $D=D_{V}N_{V}^{}/N$ and write a diffusion equation:

(14) $$\frac{\partial C(z,t)}{\partial t}=\frac{D_{V}}{N}\frac{\partial }{\partial z}\left[\left(N_{V}^{0}(z,t)+N_{V}^{-1}(z,t))\frac{\partial }{\partial z}C(z,t\right)\right],$$

which gives the *C*(*z,t*) dynamics.

## 4. Diffusion in a Model Heterostructure with a QW

### 4.1. Effect of Carrier Distribution on the QW Band Structure

Let’s first look at the energy diagram calculated for the (In,Ga)As/GaAs QW at high temperatures, in order to show that a nonuniform distribution of charge carriers actually takes place in real heterostructures. The calculation of the heterostructures’ energy spectrum was made using a nanodevice simulation tool NEXTNANO3 [59]. The parameters of the materials used in the calculation of carrier distribution and band diagrams, such as lattice constants, electron and hole effective masses of GaAs and InGaAs alloys, and the InAs/GaAs valence band offset and its temperature dependences, were taken from [55]. The strain, deformation potentials, charged carrier generation, and distribution have been taken into account in the calculations. The calculation technique was previously described in [60,61,62]. For simplicity, the exciton correction for the energy levels was neglected.

**Figure 6:**
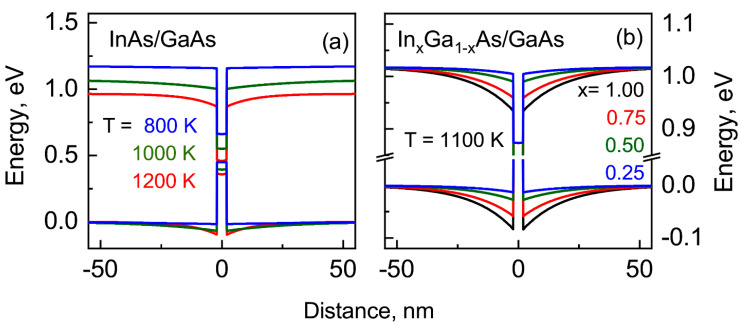
Band diagrams of heterostructures with the (In,Ga)As/GaAs QW calculated for (**a**) the InAs QW as a function of temperature and (**b**) an In*_x_*Ga*_1−x_*As QW as a function of composition *x* at *T* = 1100 K.

The band diagrams calculated for model (In,Ga)As/GaAs heterostructures containing 4 nm thickness (about eight lattice constants) QWs, taking into account equilibrium electron and hole distributions, are presented for the following: (i) the InAs QW at different temperatures, and (ii) the In*_x_*Ga_1 − *x*_ As QW at different compositions *x*, in Figure 6a and 6b, respectively. One can see the band bending near the heterojunction (In,Ga)As/GaAs, whose magnitude is increased with the increase in temperature (from a practically zero value at 800 K up to 100 meV at 1200 K). The band bending is due to the difference in the charge-carrier generation rate in the wide-gap matrix and narrow-gap quantum well and the following redistribution of these carriers in the heterostructure. It is clearly seen in Figure 6b that the band bending disappears at a fixed temperature with the decreasing QW depth as a result of the decrease in the InAs fraction in InGaAs ternary alloy.

### 4.2. Diffusivity of QW Material

In order to analyze the effect of nonuniform electron distribution on the vacancy distribution dynamics and vacancy-mediated diffusion in semiconductor heterostructures, we simulated the atomic diffusion in a simple model system with thin QWs. To model the semiconductor system with more or less realistic parameters, we took the material parameters of InAs for the QW and those of GaAs for the matrix in [55], except for ratio *m_hh_*/*m_e_*, which was taken as 50, similar to the calculation in the previous section (thus, we select here the case of Δ_1_ < 0). Using the developed model, we simulated the dynamics of gallium vacancy, which determines the diffusivity factor in heterostructures with 8·*a* thick QWs at high temperature. To take into account the effect of QW material diffusion on the concentration and distribution of thermally generated electrons, we used the iteration technique described in Appendix B. The calculated diffusivity factor was normalized to its value $D_{E}^{0}$, determined by a neutral vacancy equilibrium (at the temperature of calculation) concentration in order to emphasize the effect of charged vacancy concentration dynamics.

#### 4.2.1. Approximation of Flat Bands

**Figure 7:**
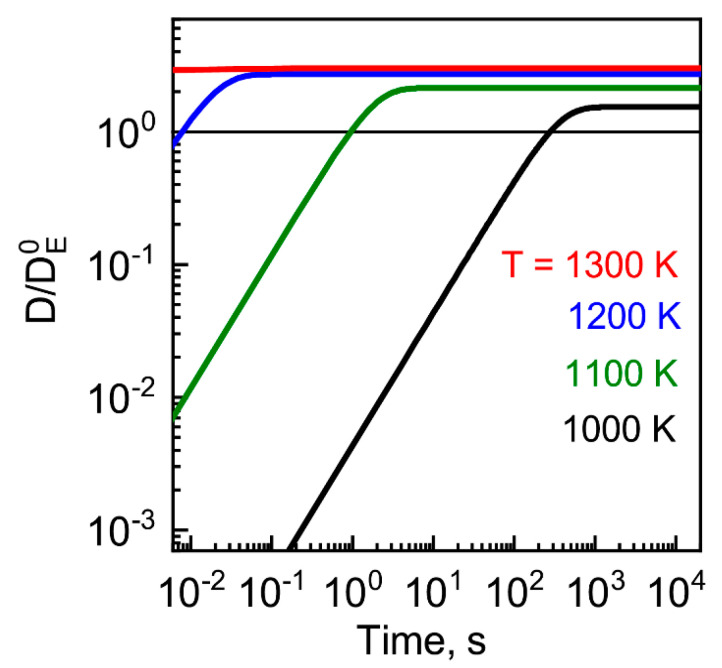
Dynamics of QW material diffusivity factor *D* vs. temperature calculated for a QW with a flat band structure. The diffusivity factor is normalized to its value $D_{E}^{0}$, determined by the equilibrium concentration of neutral vacancies.

As the first step of our study, we calculated the diffusivity factor in an approximation of a flat conduction band that corresponds to neglecting the effect of extra electron generation in the QW region on the vacancy formation, recombination, and diffusion. The dynamics of the diffusivity factor (*D*) (in this approximation, one is spatially uniform) that is calculated for different temperatures is shown in Figure 7. At low temperatures, the diffusivity factor increases with time up to saturation. The saturation level and time depend on the temperature. The first one increases and the second one decreases with the increasing temperature. This diffusivity factor saturation occurs as a result of the vacancy concentration’s reaching its equilibrium value at this temperature. Therefore, at high temperatures, the diffusivity factor quickly begins a constant. In this case, the permanence of the diffusivity factor is provided by the equilibrium concentration of charged vacancy. Thus, the approximation of a permanent diffusivity factor is well applicable in high-temperature conditions. However, even for the flat band approximation that does not take into account the effect of extra electron generation on the vacancy generation rate, there is a range of temperatures where the diffusivity factor is changed within the annealing time.

#### 4.2.2. Effect of Thermally Generated Electron Concentration and Distribution

**Figure 8:**
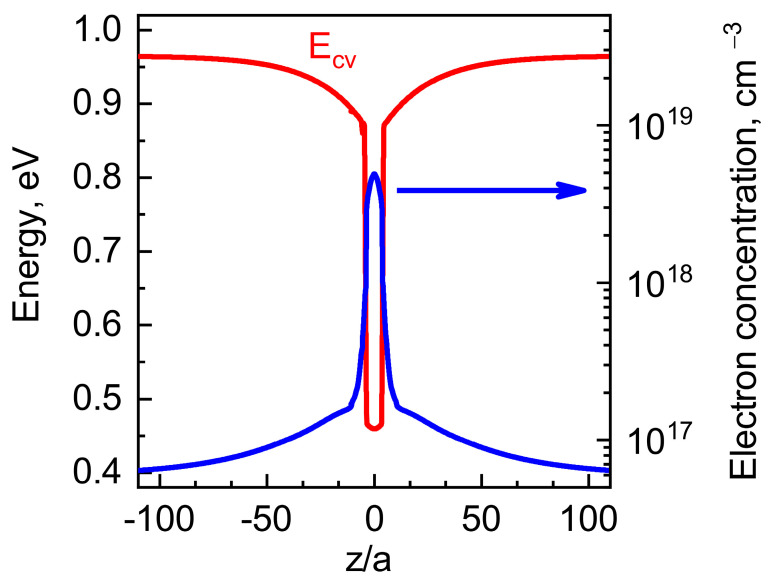
Conduction band energy (shown by red curves, left axis) and electron concentration (shown by blue curves, right axis) calculated in the heterostructure with a QW at *T* = 1200 K.

In order to demonstrate the effect of thermally generated electron concentration and spatial distribution (example shown in Figure 8), we calculated the QW material diffusivity factor dynamics. Here we need to note that vacancies and interstitial atoms evolve independently of each other after decoupling. Interstitial atom diffusivity factor *D_I_* is different from vacancy diffusivity factor *D_V_*. Thus, the spatial distributions of vacancy and the interstitial atom are also different. In a general case, when both of these defects are charged, the knowledge of dynamics for the spatial distribution of interstitial atoms is important for the determination of band-bending dynamics.

In this instance, Equation (13) should be supplemented by the expression that describes the evolution of the spatial distribution of interstitial atoms. However, in the case of neutral interstitial atoms used in our simplified model, the band bending is controlled only by the material intermixing and charged vacancy formation. To show the effect of the spatial distribution of interstitial atoms, we considered two limiting cases that simplify calculations: (i) when the interstitial atom diffusion is close to that for vacancies (in this extreme case, the spatial distributions of vacancies and interstitial atoms practically coincide) and (ii) when the interstitial atom diffusion is much faster than that of vacancies (in this extreme case, the distribution of interstitial atoms is a uniform and their concentration dynamics is determined by the processes in the matrix far from a QW). The real distribution of interstitial atoms is something between these extreme cases.

**Figure 9:**
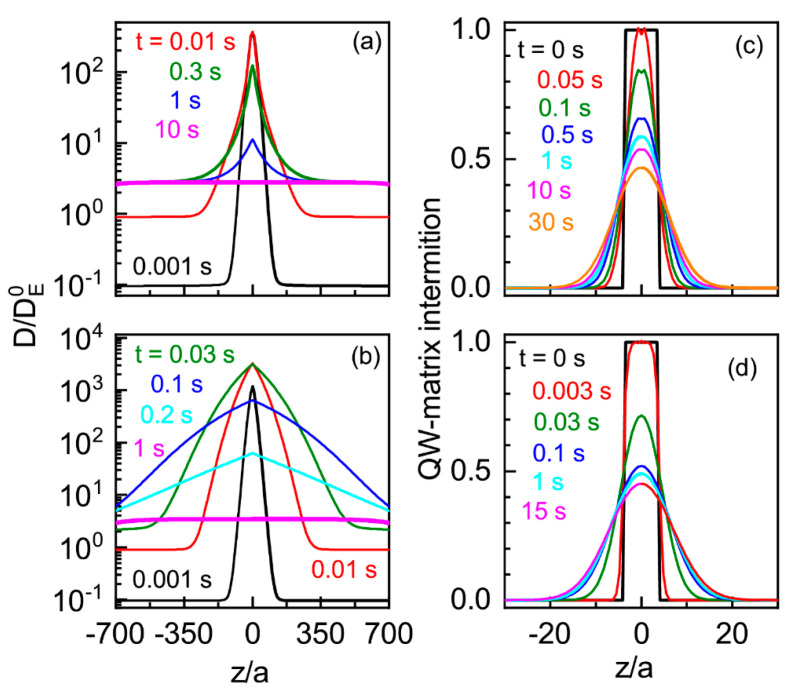
Dynamics of diffusivity factor (**a**,**b**) diffusion profiles of the quantum well (**c**,**d**). The diffusivity factor is normalized to its value $D_{E}^{0}$, determined by the equilibrium concentration of neutral vacancies: (**a**,**b**) the case of the diffusion of interstitial atoms is close to that for vacancies; (**b**,**d**) the diffusion of interstitial atoms is much faster than that of vacancies.

**Figure 10:**
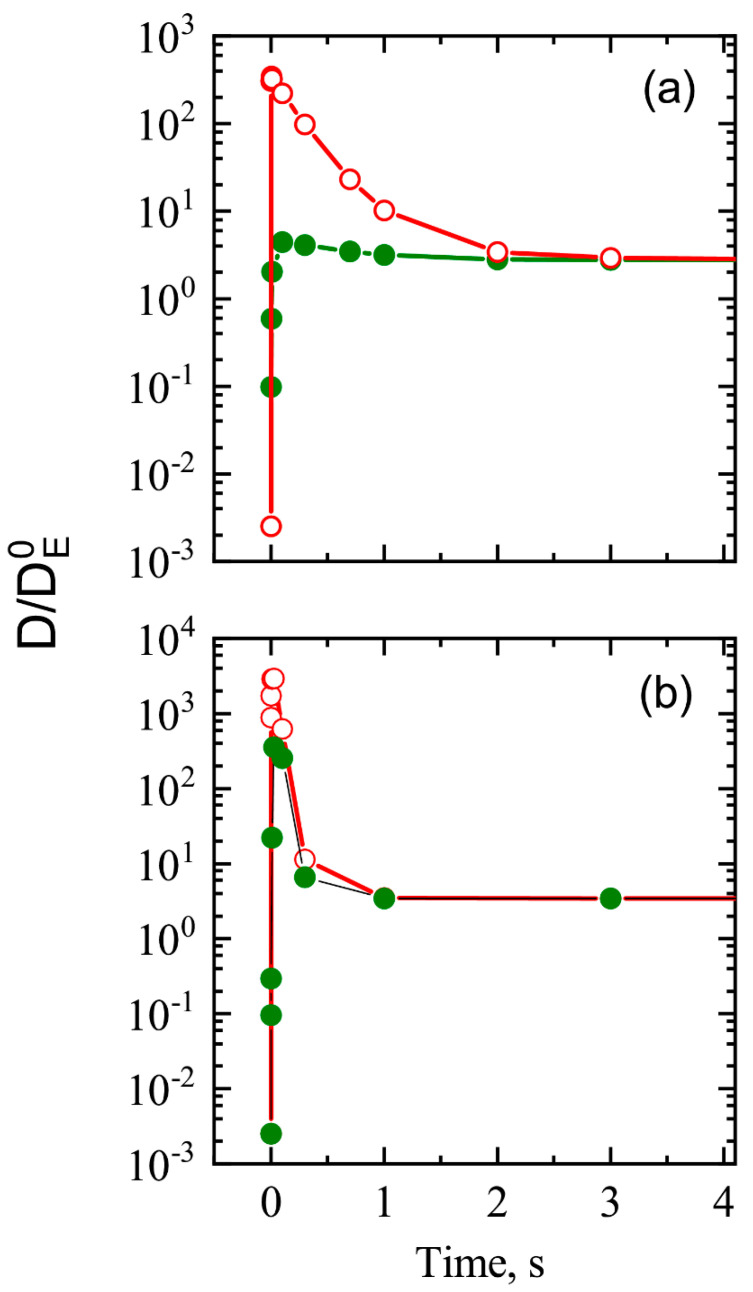
Dynamics of diffusivity factor calculated at 1200 K for the heterostructure with a QW at the spatial position near the heterointerface (opened red circles) and at spatial position shifted from heterointerface in 30 lattice constants (filled olive circles). (**a**) The case of interstitial atom diffusion is close to that for vacancies. (**b**) The case of interstitial atom diffusion is much faster than that of vacancies. The diffusivity factor is normalized to its value $D_{E}^{0}$, determined by the equilibrium concentration of neutral vacancies.

The spatial distribution dynamics of the diffusivity factor, calculated for the QW at a temperature of 1200 K in both of these extreme cases, is presented in Figure 9. Actually, the spatial distribution of the diffusivity factor is governed by (i) the vacancy generation around the QW and (ii) the vacancy diffusion that removing vacancies from the QW/matrix heterojunction region. One can see that the local increase in electron concentration results in a very strong change in the diffusivity factor, which is proportional to the vacancy concentration ($D(t)\sim N_{V}(t)/N$). However, the excess of electrons that appears at a high temperature in the region around the QW very quickly disappears thanks to ”a negative feedback” provided by the increase in the QW band gap and the formation of charged vacancies, which captured the electrons (in the case of our calculation, the last is dominated). The diffusivity factor becomes uniform in space. For the case when the interstitial atom diffusion is much faster than that of vacancies, the diffusivity factor quickly and more strongly increases, but it more quickly decreases to the equilibrium value (see Figure 10), because the feedback efficiency increases with the charged vacancy concentration. That is a result of the local decrease in the interstitial atom concentration near QWs, which shift the result of the ratio for the vacancy generation and recombination rates (it also results in the spread of the space region, where *D* deviates from the value determined by the vacancy concentration in the matrix).

In conclusion of this section, let us present the most important findings of the modeling. (1) A spatially uniform diffusivity factor distribution in heterostructures with QWs is realized at high temperatures, when the vacancy generation quickly leads to the set of their equilibrium (at this temperature) concentration. (2) There are ranges of annealing temperatures and times, for which the diffusivity factor in heterostructures can be spatially inhomogeneous and changes with the annealing time. The spatial diffusivity factor distribution dynamics is a result of the redistribution for the density of thermally generated electrons in the QW range and that leads to a spatially nonuniform vacancy generation rate and diffusion.

The simplicity of the model, together with the uncertainty in parameters governed by the vacancy generation and migration (see Table 1), does not provide a quantitative description of the experiments. However, one can find a confirmation for our conclusions in available experimental data. In [63], two distinct diffusion regimes, specifically fast initial diffusion and steady-state diffusion, were experimentally revealed at the high-temperature annealing of heterostructures with (In,Ga)As/GaAs QWs. In heterostructures containing In*_x_*Ga*_1−x_*As/GaAs QWs, a monotonic increase in the indium diffusivity factor with an increase in the indium arsenide fraction, in the range from *x* = 0.05 to *x* = 0.2 (i.e., with decreasing the band gap that leads to the increase in the electron concentration in the QW region), was experimentally demonstrated for different fixed annealing temperatures [64]. Different thermo-activation energy values of the indium diffusivity factor, obtained in (In,Ga)As/GaAs heterostructures containing quantum wells and quantum dots with different ternary alloy compositions (1.1 eV [50], 1.23 eV [12], 3.0 eV [63], 3.4 eV [21]), can also be induced by different thermally generated electron concentrations.

Finally, a high concentration of thermally generated charge carriers is high in semiconductor heterostructures with narrow band-gap QWs. With an increase in the band gap, the concentration of equilibrium charge carriers and their influence on the atomic diffusion processes will exponentially decrease.

## 5. Conclusions

We demonstrated that the nonuniform spatial distribution of vacancy concentration can appear at high temperatures in semiconductor heterostructures with an undoped quantum well as a result of strong dependence in the vacancy formation rate on the local charge-carrier density. A theoretical model of vacancy-mediated diffusion in semiconductor heterostructures with QWs at high-temperature annealing was developed. The model took into account the dynamics of the spatial distributions of thermally generated charged carriers that affect the spatial vacancy distribution dynamics. We show that in some temperature and time regions, which depend on semiconductor heterosystem parameters, diffusivity factors in such heterosystems can be nonuniform in space and change with the annealing time. The spatial nonuniformity is a result of nonuniformity in the charged vacancy generation that stems from a gradient of equilibrium carrier concentration, which appears due to differences in the electron-hole generation rate in the regions with different band gaps. The change with time stems from two reasons: (i) a change in the vacancy concentration from the background to equilibrium level and (ii) a change in the local electron concentration with a change in the band-gap energy owing to the material’s intermixing and with a charged defect formation. The spatial nonuniformity and the dynamics of diffusivity were demonstrated for narrow band-gap QWs by computational modeling. In fact, the phenomenon revealed in our study is very general and takes place at high temperatures for the formation and migration of any charged defects in any low-dimensional structures constructed from different semiconductor materials.

The features of vacancy-mediated diffusion in undoped semiconductor heterostructures during high-temperature annealing revealed in this work can be used to correct the parameters of QWs and QDs on the basis of II-VI and III-V compounds, such as ZnS/ZnSSe or InGaAs/AlGaAs, which are widely used in modern optoelectronics devices. The possibilities for describing the vacancy formation dynamics in heterostructures are especially interesting for making the optimal choice of post-growth rapid thermal annealing conditions that allow the reduction of the strain gradients and allow the modification of the band-gap structure and exciton oscillator strength in such heterostructures.

## Figures and Tables

**Table 1:** Parameters of gallium vacancy in GaAs: formation enthalpy $H_{f}^{0}$ and entropy $S_{f}^{}$, migration enthalpy $H_{m}^{0}$ and entropy $S_{m}^{}$, and electron ionization energy in different charged states $E_{V}^{-1}$, where ν is the Debye frequency.

Parameter	Value
$H_{f}^{0}$ (eV)	2.8 [53], 3.2 [26], 3.7 [54]
$H_{m}^{0}$ (eV)	0.8 [50], 1.8 [48], 2.7 [52], 3.3 [47]
$S_{f}^{}$	7.3 × *k* [53]
$S_{m}^{}$	11.3 × *k* [47]
ν (Hz)	10^13^ [44]
$E_{V}^{-1}$	0.13 × $E_{g}^{}$ [23,26]

**Figure A1:**
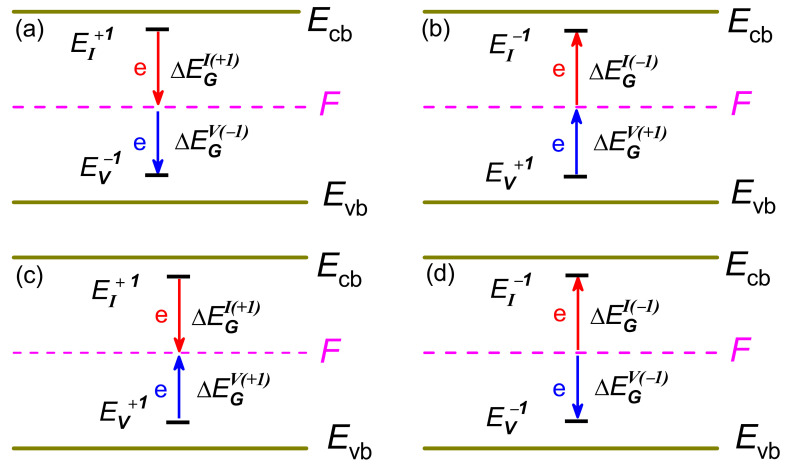
Schematic energy diagram for the Frenkel pair containing (**a**) the once negatively charged vacancy and the once positively charged interstitial atom, (**b**) the once positively charged vacancy and the once negatively charged interstitial atom, (**c**) the once positively charged vacancy and the interstitial atom, and (**d**) the once negatively charged vacancy and the interstitial atom. The arrows show the change (up = increase and down = decrease) in the electronic system energy released at the charged defect formation.

## Data Availability

Data are available from the authors on request.

## Appendix A

Let both defects in a Frenkel pair have a charge. We consider the change in the probability of such pair formation relative to that for a pair of neutral defects.

Case (0): The Frenkel pair contains neutral defects only. The formation probability is proportional to *A^0^*exp(−*H*_A_/*kT*), as it is shown in Section 2.1.

Case (1): When the Frenkel pair of a neutral interstitial atom and *j*-negatively charged vacancy is formed *j* electrons are captured in the vacancy levels. As it was shown in the main text of the article, in this case, the pre-exponential factor of Case (0) is multiplied by the probability of founding *j* electrons at the spatial point, where the Frenkel pair is formed $A^{\left(-j\right)}=A^{0}{\left(n/N_{ce}\right)}^{2j}$ and the formation energy decreases $H_{A}^{V(-j)}=H_{A}^{0}+\Delta E_{G}^{V(-j)}$ by the sum of the differences between the Fermi level and the ionization energy of the vacancy that captured 1, 2, …, and *j* electrons. $\Delta E_{G}^{V(-j)}=\sum_{i=1}^{j}\left(E_{V}^{i}-F\right)$.

Case (2): When the Frenkel pair is formed by a negatively charged vacancy and a positively charged interstitial atom (typical of the cation sublattices of III-V compounds), the vacancy captures *j* electrons and the interstitial atom gives *k* electrons to the crystal during the formation. Pre-exponential factor $A_{I(+k)}^{V(-j)}$ is the same as in Case (1), and the energy of pair formation is changed in the following manner:$H_{A}^{V(-j)I(+k)}=H_{A}^{0}+\Delta E_{G}^{V(-j)}+\Delta E_{G}^{I(+k)}$ (where $\Delta E_{G}^{I(+k)}=\sum_{i=1}^{k}\left(F-E_{I}^{i}\right)$ is the sum of the differences between the position of the *k*-electron level of the interstitial atom and the Fermi level). The change in electron system energy at the formation of the once negatively charged vacancy and once positively charged interstitial atom is schematically shown in Figure A1a. Therefore, the probability of the formation of a Frenkel pair containing a *j*-negatively charged vacancy and a *k*-positively charged interstitial atom, in the general case, can be written as follows:

$$A_{I(+k)}^{V(-j)}\exp\left[-\frac{H_{A}^{0}+\sum_{i=1}^{j}\left(E_{V}^{i}\right)-\sum_{i=1}^{k}\left(E_{I}^{i}\right)+(k-j)F}{kT}\right].$$

In this scenario, schematically shown in Figure A1a, the activation energy of the pair formation will decrease relative to that for a pair of neutral defects, similar to the simple case of a neutral interstitial atom in the Frenkel pair used in the main text.

Case (3): The Frenkel pair is formed by a positively charged vacancy and a negatively charged interstitial atom (typical of the anion sublattices of III-V compounds). Here the vacancy ejects *j* electrons, and the interstitial atom captures *k* electrons in the crystal during the formation. The expressions describing the process are very similar to Case (2). The pre-exponential factor similar to Case (1) has to be multiplied by the probability of founding *k* electrons at the spatial point, where the Frenkel pair is formed $A_{I(-k)}^{V(+j)}=A^{0}{\left(n/N_{ce}\right)}^{2k}$, and the additional term to $H_{A}^{0}$ is $\Delta E_{G}^{I(-k)}+\Delta E_{G}^{V(+j)}=\sum_{i=1}^{j}\left(F-E_{V}^{i}\right)+\sum_{i=1}^{k}\left(E_{I}^{i}-F\right)$. Thus, the formation probability of the Frenkel pair containing a *j*-positively charged vacancy and *k-*negatively charged interstitial atoms, in the general case, could be written as follows:

$$A_{I(-k)}^{V(+j)}\exp\left[-\frac{H_{A}^{0}-\sum_{i=1}^{j}\left(E_{V}^{i}\right)+\sum_{i=1}^{k}\left(E_{I}^{i}\right)+(j-k)F}{kT}\right].$$

The changing of the electron system energy at the formation of the once positively charged vacancy and the once negatively charged interstitial atom are schematically shown in Figure A1b. Here the effective activation energy of the pair formation will increase relative to that for the pair of neutral defects.

In the hypothetical cases, when the Frenkel pair is formed by defects with the same charge sign, for a positive (negative) case, the pre-exponential factor equals $A_{I(+k)}^{V(+j)}=A^{0}$ ($A_{I(-k)}^{V(-j)}=A^{0}{\left(n/N_{ce}\right)}^{2(k+j)}$) and the term additional to $H_{A}^{0}$ equals $\Delta E_{G}^{I(+k)}+\Delta E_{G}^{V(+j)}=\sum_{i=1}^{j}\left(F-E_{V}^{i}\right)+\sum_{i=1}^{k}\left(F-E_{I}^{i}\right)$ ($\Delta E_{G}^{I(-k)}+\Delta E_{G}^{V(-j)}=\sum_{i=1}^{j}\left(E_{V}^{i}-F\right)+\sum_{i=1}^{k}\left(E_{I}^{i}-F\right)$), respectively. In these cases, the energy balance is determined by the ratio of the difference in the binding energies of the electrons to the Fermi level at the vacancy and the interstitial atom. The examples for the once positively charged vacancy and the negatively charged vacancy and those for the interstitial atom are schematically shown in Figure A1c,d, respectively.

## Appendix B

For simplicity, we used the iteration technique of the calculation. In the first step, the band diagram of QW is calculated for the initial material distribution *C*(*z*, *0*) = 1 for −2 ≤ *z* ≤ 2 nm and 0 for |*z*| > 2 nm using a NextNano3 [59] simulation suite. The *E_cb_*(*z*, 0) spatial distribution and Fermi-level energy, *F*, were obtained for the calculated QW energy spectrum. The dynamics of the *G*(*z*, *t*), *N_V_*(*z*, *t*), and *C*(*z*, *t*) parameters are calculated by using the equations of the system (13) with the fixed spatial distribution *E_cb_*(*z*, *t*) = *E_cb_*(*z*,0) till the C(*z* = 0, *t*) value becomes equal to *X*_1_ (at time *t*_1_). At the second iteration, the band diagram of the QW is recalculated for material distribution *C*(*z*, *t*_1_) in order to obtain the spatial distributions *E_cb_*(*z*, *t*_1_). The spatial distribution of *C*(*z*,t_1_) and *N*_V_(*z*, *t*_1_) are taken as the initial conditions, and *E_cb_*(*z*, *t*) are again fixed at *E_cb_*(*z*, *t*_1_). The dynamics of all parameters are calculated till *C*(*z* = 0, *t*) becomes equal to *X*_2_ (at time *t*_2_). Everything is repeated in the next iterations. Thus, the spatial distributions *E_cb_*(*z*, *t*) used in our calculation do not change continuously in time, but instead, they are fixed in a time range of *t_k_*_−1_ < *t* < *t_k_*, till the material concentration in the QW center lies in the range of *X_k−_*_1_ < *C*(0, *t*) < *X_k_*. We used a row of *X_k_* = 1 − 0.05 × *k* in the calculations.
